# Household Food Insecurity and the Association with Cumulative Biological Risk among Lower-Income Adults: Results from the National Health and Nutrition Examination Surveys 2007–2010

**DOI:** 10.3390/nu12051517

**Published:** 2020-05-23

**Authors:** Cindy W. Leung, Megan S. Zhou

**Affiliations:** Department of Nutritional Sciences, School of Public Health, University of Michigan, Ann Arbor, MI 48109, USA; zhoumeg@umich.edu

**Keywords:** food insecurity, allostatic load, biological risk, chronic stress, National Health and Nutrition Examination Surveys

## Abstract

Household food insecurity has been associated with adverse health outcomes; however, the mechanisms underlying these associations are not well-defined. Using data from 5005 adults from the 2007–2010 National Health and Nutrition Examination Surveys (NHANES), we examined associations between household food insecurity and cumulative biological risk, a measure of the body’s physiological response to chronic stress. Household food security was assessed using the 18-item Household Food Security Survey Module. Marginal food security refers to 1–2 positive responses, and food insecurity refers to ≥3 positive responses. The cumulative biological risk scores were calculated based on the distributions of ten biomarkers from the cardiovascular, metabolic, and immune systems. Elevated biological risk was defined as a risk score of ≥3. Multivariable regression models were used to examine associations between food security and cumulative biological risk scores, adjusting for sociodemographic characteristics. After multivariable adjustment, food insecurity was associated with a 0.14-unit higher cumulative biological risk score (95% CI 0.05–0.22, *p*-trend = 0.003) and higher odds of elevated biological risk (OR 1.20, 95% CI 1.05–1.37, *p*-trend = 0.003). These associations differed by gender. Among women, food insecurity was associated with 0.30-unit higher cumulative biological risk score (95% CI 0.14–0.45, *p*-trend = 0.0004) and higher odds of elevated biological risk (OR 1.61, 95% CI 1.29–2.00, *p*-trend < 0.0001). These associations were not observed in men. Women experiencing food insecurity demonstrated elevated levels of biological risk. These findings support the hypothesis that food insecurity may be associated with women’s chronic health outcomes through the pathway of chronic stress. Further research is needed to understand why these associations were not observed in men.

## 1. Introduction

Food insecurity, defined as inadequate access and availability of food due to a lack of monetary resources, has persisted in the United States since its routine measurement in national population surveys in the 1990s [1]. In 2018, it was estimated that 14.3 million households or 11.1% of U.S. households experienced food insecurity during the year [1]. Household food insecurity has been associated with numerous physical and mental health outcomes among low-income adults, including higher levels of obesity [2,3,4], hypertension [5], diabetes [6,7] and poorer diabetes management [8,9], metabolic syndrome [10], lower cognitive function [11,12], depression [13,14], and poorer overall health [15,16]. The mechanisms underlying these associations have not been fully elucidated; however, a number of studies have demonstrated associations between household food insecurity and modifiable health behaviors, such as poorer diet quality [17,18,19], lower levels of physical activity [20], higher rates of smoking [21,22,23], and poorer sleep outcomes [24,25]. Several researchers have also alluded to the role of chronic stress in explaining the observed associations [14,26,27,28,29,30], though none of these studies have been able to test the association between food insecurity and chronic stress directly.

Chronic stress refers to the repeated activation of major body systems in response to external stimuli perceived to be threatening [31]. In response to a stressor, the hypothalamic–pituitary–adrenal (HPA) axis and the sympathetic–adrenal–medullary (SAM) systems are activated, triggering a release of hormones and cytokines that act on multiple organ systems [31,32]. The concept of allostatic load has been used to describe how repeated exposure and prolonged response to stress can dysregulate these organ systems, resulting in “wear and tear” on the body over time [32]. Operationally, there is no single, gold-standard approach for measuring allostatic load, though allostatic load scores often include a combination of biomarkers from neuroendocrine, immune, metabolic, and cardiovascular systems—all known to be affected by the secretion of stress hormones and inflammatory markers [33]. Thus, allostatic load is theorized to represent the body’s cumulative physiological response to chronic stress and to account for individual variability in the appraisal of different stress exposures, which may be more relevant to predicting subsequent health outcomes than the perception of stress [34].

Prior research has explored the role of the allostatic load as a framework through which to understand health disparities. Greater socioeconomic adversity [35,36,37,38] and minority race/ethnicity [39,40,41] have both been associated with elevated allostatic load in numerous studies. Household food insecurity is both a form of socioeconomic adversity and an issue with relatively high prevalence in low-income and minority racial/ethnic households [1]. However, its relation to allostatic load has only been explored in one study [42]. In an analysis of 733 Puerto Rican adults residing in Boston, food insecurity was associated with greater dysregulation of the neuroendocrine and inflammatory systems, but not total allostatic load [42]. In order to understand the pathways connecting food insecurity and adverse health outcomes, more research is needed to examine this association in a larger and more representative sample of adults.

In the present study, neuroendocrine markers are important components of allostatic load measurement but were unavailable in the National Health and Nutrition Examination Surveys (NHANES). Thus, we created a score to represent a cumulative biological risk, guided by the allostatic load framework. The objective of the present study was to examine the association between household food insecurity and cumulative biological risk in a national sample of adults. We hypothesized that adults who experienced more severe household food insecurity would demonstrate greater cumulative biological risk.

## 2. Materials and Methods

### 2.1. Study Population

Administered by the National Center for Health Statistics, NHANES is an ongoing, multistage survey designed to be representative of the civilian, noninstitutionalized U.S. population. NHANES collects information on demographic indicators and health outcomes through interviews, in-person examinations, and laboratory testing. Data from 2007 to 2008 and from 2009 to 2010 were combined in the present study, representing the most recent years in which all markers of cumulative biological risk were routinely collected (e.g., C-reactive protein was not assessed in NHANES 2011–2012 or 2013–2014). The analytic population was further restricted to adults between the ages of 20–65 and with household incomes ≤300% of the federal poverty level (FPL). An income threshold of 300% FPL was chosen because household food insecurity is relatively uncommon among households with incomes >300% FPL, and inclusion of a lower-income sample reduces the potential for confounding by socioeconomic status, as has been done in prior studies [6,17,43]. Pregnant women were also excluded, as body mass index (BMI) was one of the markers of biological risk. The analytic sample included 5005 adults.

### 2.2. Food Security Status

The primary exposure of interest was household food security, measured using the 18-item U.S. Household Food Security Survey Module [44]. Questions were asked in three stages and attribute related experiences or behaviors to insufficient resources to buy food over the past 12 months. A score was created by summing the affirmative responses of the 18 questions, with higher scores indicating more severe food insecurity. Food security was defined as 0 affirmative responses, meaning the household had no indicators of insufficient food access. Marginal food security was defined as 1–2 affirmative responses and refers to mild indicators of insufficient food access such as anxiety over the food supply. Food insecurity was defined as three or more affirmative responses and refers to multiple indicators of insufficient food access, including reducing the quality and the variety of the amount of food consumed by at least one member of the household. Food insecurity refers to the combined categories of low food security (i.e., 3–5 affirmative responses in households without children or 3–7 affirmative responses in households with children) and very low food security (i.e., 6–10 affirmative responses in households without children or 8–18 affirmative responses in households with children). Food insecurity categorizations and definitions are in accordance with the U.S. Department of Agriculture [44].

### 2.3. Cumulative Biological Risk

To assess cumulative biological risk, we included the following ten biomarkers: (1) systolic blood pressure, (2) diastolic blood pressure, (3) body mass index (BMI), (4) glycohemoglobin, (5) total cholesterol, (6) high-density lipoprotein (HDL) cholesterol, (7) total/HDL cholesterol ratio, (8) C-reactive protein, (9) albumin, and (10) estimated glomerular filtration rate (eGFR). The selection of biomarkers for the present study was guided by the availability of data within NHANES and on the basis of previous research [45,46,47,48]. Systolic blood pressure, diastolic blood pressure, total cholesterol, HDL cholesterol, and pulse represented the cardiovascular system. BMI, glycohemoglobin, albumin, and eGFR represented the metabolic system. C-reactive protein represented the immune system.

Each biomarker was categorized using clinically-relevant guidelines for low-risk, moderate-risk, and high-risk categories [47]. The cut-points used were: (1) systolic blood pressure: <120 mmHg, 120–<150 mmHg, and ≥150 mmHg; (2) diastolic blood pressure: <80 mmHg, 80–<90 mmHg, and ≥90 mmHg; (3) BMI: <25 kg/m^2^, 25–<30 kg/m^2^, ≥30 kg/m^2^; (4) glycohemoglobin: <5.7%, 5.7–<6.5%, and ≥6.5%; (5) total cholesterol: <200 mg/dL, 200–<240 mg/dL, ≥240 mg/dL; (6) HDL cholesterol: ≥60 mg/dL, 40–<60 mg/dL, and <40 mg/dL; (7) total/HDL ratio: <5; 5–<6, ≥6; (8) C-reactive protein: <1 mg/L, 1–<3 mg/L, and ≥3 mg/L; (9) albumin: ≥3.8, 3.0–<3.8, and <3.0; and (10) eGFR: ≥60 mL/min/1.73 m^2^, 30–<60 mL/min/1.73 m^2^, and <30 mL/min/1.73 m^2^. Each biomarker was scored as zero points for low risk, 0.5 points for moderate risk, and one point for high risk. Adults who reported taking medication for high blood pressure, high cholesterol, or diabetes were also assigned to the high-risk groups for systolic and diastolic blood pressure, total cholesterol, and glycohemoglobin, respectively. A cumulative biological risk score was then created as the sum of the risk scores across the ten components, ranging from 0 (lowest) to 10 (highest). Similar to prior studies [45,46], elevated biological risk was defined as a score ≥3. Both continuous and dichotomous cumulative biological risk scores were examined as primary outcomes.

### 2.4. Study Covariates

Sociodemographic covariates were selected as variables hypothesized to be joint predictors of the association between food insecurity and cumulative biological risk, guided by the prior literature. These included age (continuous), gender, race/ethnicity (Non-Hispanic White, Non-Hispanic Black, Hispanic, Other), educational attainment (<12 years, high school graduate or equivalent, some college, or college graduate), household income relative to the federal poverty line (continuous), and marital status (married or living with a partner, never married, or separated, widowed, or divorced).

### 2.5. Statistical Analysis

Complex sampling weights for the mobile examination center were recalculated and used to account for different sampling probabilities and participation rates across the four-year period. Sampling weights were used in all subsequent analyses. Differences in sociodemographic characteristics by household food security were compared using univariate regression for continuous variables and χ^2^ tests for categorical variables. Next, the distributions in individual biomarkers by household food security were compared using univariate regression and χ^2^ tests. Multivariable linear and logistic regression models were used to examine associations between food security and cumulative biological risk. Differences in these associations by gender were evaluated by testing the significance of the interaction terms between household food security and gender on the outcomes. Models were first adjusted for age and gender, and second for all other sociodemographic covariates (race/ethnicity, educational attainment, household income, and marital status). In all models, age and household income were modeled as linear and quadratic terms to allow for a curvilinear relationship with cumulative biological risk. In a sensitivity analysis, we further examined the associations between the household food security and cumulative biological risk using a four-category household food security variable. Statistical tests were two-sided, and significance was considered at *p* < 0.05. Statistical analyses were performed with SAS 9.3 (SAS Institute Inc., Cary, NC, USA).

## 3. Results

In the analytic population of 5005 adults, 59.3% were food-secure, 14.4% were marginally food secure, and 26.2% were food-insecure. Table 1 shows the differences in the sociodemographic characteristics by household food security. Compared to food-secure adults, marginally food-secure and food-insecure adults were more likely to be of younger age, of minority race/ethnicity background, have lower educational attainment, have lower household income, and were more likely to be never married, or separated, divorced, or widowed.

Bivariate comparisons of biomarkers comprising cumulative biological risk and household food security are shown in Table 2. Compared to food-secure adults, marginally food-secure and food-insecure adults were more likely to have higher mean glycohemoglobin (*p* = 0.01), C-reactive protein (*p* = 0.005), and albumin (*p* = 0.0002). Significant differences were also evident for some risk categories. Marginally food-secure adults were more likely to be at moderate- or high-risk for systolic blood pressure (*p* = 0.04), and both marginally food-secure and food-insecure adults were more likely to be at moderate- or high-risk for HDL cholesterol (*p* = 0.05). There were no other significant bivariate associations between individual biomarkers and food security status.

Table 3 shows the associations between household food security and cumulative biological risk. Food insecurity was associated with a 0.22-point greater cumulative biological risk score (95% CI 0.11–0.32, *p*-trend = 0.0002), which remained significant after multivariate adjustment (β = 0.14, 95% CI 0.05–0.22, *p*-trend = 0.003). Although the associations between marginal food security and cumulative biological risk and between food insecurity and cumulative biological risk both appeared stronger in women than in men, the interaction was not statistically significant (*p*-interaction = 0.09).

When examining elevated biological risk (score ≥3), food insecurity was associated with higher odds of elevated biological risk (OR 1.20, 95% CI 1.05–1.37, *p*-trend = 0.003), after adjusting for sociodemographic characteristics. This association was significantly modified by gender (*p*-interaction = 0.03). Among women, food insecurity was associated with elevated biological risk (OR 1.61, 95% CI 1.29–2.00, *p*-trend < 0.0001). Among men, no association was observed between food insecurity and elevated biological risk (OR 0.93, 95% CI 0.72–1.20, *p*-trend = 0.70). A sensitivity analysis using a four-category household food security variable showed similar results with cumulative biological risk scores and elevated biological risk (Supplemental Table S1).

## 4. Discussion

In this national sample of lower-income adults, food insecurity was significantly associated with elevated biological risk in women. Significant associations were also observed between marginal food security and food insecurity and higher cumulative biological risk scores, supporting the notion that marginal food security is similar to food insecurity with respect to adverse health risks [30,49]. These findings suggest that food insecurity, and potentially even experiences of marginal food security are associated with the dysregulation of the major body systems in women [31,50].

To date, only one other study has examined the association between food insecurity and allostatic load. Among 733 Puerto Rican adults in the Boston Puerto Rican Health Study (BPHC), McClain and colleagues found that food insecurity was associated with greater dysregulation of the neuroendocrine and immune systems over a five-year follow-up period, but not with the total allostatic load [42]. The differences in these findings may be due to BPHC study participants being older and already having higher burden of chronic disease at baseline than the general NHANES population, and the lack of neuroendocrine markers within NHANES to investigate this specific association.

The results of the present study also highlight differences in the associations between food insecurity and cumulative biological risk between men and women. Although it is unclear why the associations were significant among women and not significant among men, prior research on the connections between food insecurity and stress may provide insight into these differences. The development of the early Radimer/Cornell hunger scale drew primarily from women’s experiences of household food insecurity and demonstrated that one of the earliest indicators was worrying about the household food resources being depleted [51]. This item, now in the U.S. Household Food Security Survey Module, is consistently endorsed by the vast majority of food-insecure households [1]. Qualitative research studies exploring the lived experiences of women have also expanded our understanding of the stressful experience of food insecurity. In a study of predominantly mothers from Quebec City, participants described the “psychological suffering” of food insecurity, as feelings of powerlessness, guilt, shame, feelings of inequity, and fears of being judged or labeled [29]. Mothers from another study in Philadelphia and Minneapolis discussed the continual trade-off between food and other basic necessities due to limited financial resources, characterizing their psychological response as sadness, frustration, resignation, worry and fear, and shame [52]. In quantitative studies of pregnant women, food insecurity has been related to higher reported levels of perceived stress, disordered eating behaviors, trait anxiety, and depressive symptoms, and lower self-esteem and mastery [53,54]. Furthermore, a systematic review on social position, stress, and obesity-related risk factors concluded that women not only perceive stress more strongly but also exhibit a greater physiological response to social stressors when compared to men [55]. At the present time, more qualitative and quantitative research is needed to better understand how men’s psychological and physiological responses to food insecurity may differ from the responses of women and how those differences may translate into subsequent implications for health.

Although the present study did not include dietary intake as a mediator or outcome, the inverse association between food insecurity and diet quality has been well-established in prior studies. In a study of food pantry clients in Connecticut, food insecurity was associated with a lower likelihood of consuming fruits, vegetables, and fiber [56]. Another study in Texas found that urban and rural adults experiencing food-related hardship were more likely to consume sugar-sweetened beverages [57]. Within NHANES, a previous analysis showed inverse associations between household food insecurity and adult’s dietary quality, as indicated by the lower scores on the Healthy Eating Index and the Alternate Healthy Eating Index [17]. The results from the present study on household food insecurity and some biomarkers of cumulative biological risk, e.g., glycohemoglobin, HDL cholesterol, and systolic blood pressure, may be driven, in part, by differences in dietary quality rather than chronic stress. However, the chronic stress and dietary pathways stemming from food insecurity are not mutually exclusive, and several studies have demonstrated how chronic stress could also alter eating behaviors to negatively impact dietary quality. Chronic stress activates the hypothalamus–pituitary–adrenal (HPA) axis, which triggers a cascade of hormones, leading to the release of cortisol [32,58]. Cortisol stimulates food intake, particularly foods high in fat and sugar, and can lead to excessive caloric intake and cardiometabolic disease over time [59,60]. When food is available, food-insecure individuals may overeat not simply in response to the physical sensation of hunger, but as physiological and behavioral coping strategies to chronic stress. In a qualitative study by Tester and colleagues, food-insecure parents discussed disordered eating habits observed in their children, including binge eating, hiding food, and night-time eating behaviors not discussed by food-secure parents [61]. To date, there have been few studies on the associations between household food insecurity and disordered eating among adults, with most research limited to children [62,63] and pregnant women [54,63]. Further research, particularly using longitudinal study designs and robust measurement of food insecurity, chronic stress, diet quality and eating behaviors, and multiple systems comprising allostatic load, are needed to better understand the relationship between food insecurity and allostatic load and elucidate the pathways of chronic stress and dietary intake in the general population.

The results of this study have potential clinical and policy implications. The finding that even marginal food security was associated with higher cumulative biological risk scores among women is consistent with research showing that children in households with marginal food security exhibit poorer health and developmental outcomes than children in food-secure households [49]. Screening of food insecurity in health care settings using the validated Hunger Vital Sign measure can help identify adults with marginal food security for the referral to community food programs and social services [64]. Economic and nutrition programs and policies aimed at improving food security should also ensure that they are reaching populations with marginal food security to ameliorate any adverse health outcomes related to anxiety over household food resources and milder indicators of food insecurity that precede behavioral adaptations.

The primary limitation of this study is the cross-sectional design, which limits the ability to make causal inferences about the findings. Although we restricted the analytic sample to adults with household incomes ≤300% of the federal poverty level and further adjusted for household income in statistical analyses, we cannot rule out the potential for residual confounding by income or other proxies of socioeconomic status, which are known to have salient relationships with health behaviors, physical health, and mental health [65]. Relatedly, we cannot exclude the potential for reverse causation, where elevated biological risk might lead to increased health care costs, subsequently influencing household food security. Another limitation is the assessment of household food security in NHANES, which occurs over a 12-month period. By aggregating over the past year, our understanding is limited as to whether experiences of food insecurity were episodic or chronic. Prior research suggests food-insecure individuals may exhibit disordered eating behaviors corresponding to a monthly cycle of when food is plentiful or scarce [61,66]. How food insecurity-induced disordered eating is associated with cumulative biological risk is unknown given the long period over which food insecurity indicators are measured. Further, no information is available on the history of food insecurity in the family, as cumulative experiences of food insecurity experienced before the study window may have also contributed to biological risk.

Another important limitation of the NHANES dataset is that it lacks neuroendocrine markers to better measure allostatic load. Further research is needed to understand how food insecurity is associated with neuroendocrine dysregulation and the primary system of allostatic load to better understand its relationship with chronic stress.

## 5. Conclusions

Understanding the mechanisms underlying food insecurity and adverse health outcomes is critical to designing effective interventions to reduce socioeconomic and health disparities. The findings of the present study show higher cumulative biological risk scores among marginally food-secure and food-insecure women, providing additional evidence to suggest even mild experiences of food insecurity may affect physical and mental health outcomes through chronic stress. Further research is needed to understand why these associations were not observed in men and to better elucidate the role of food insecurity in promoting chronic stress independent of other forms of socioeconomic adversity in women.

## Figures and Tables

**Table 1:** Household food security and sociodemographic characteristics of 5005 adults (20–65 year) with household incomes ≤300% of the federal poverty level: National Health and Nutrition Examination Surveys 2007–2010.

	Food Secure (*n* = 2599)	Marginal Food Secure (*n* = 819)	Food Insecure (*n* = 1587)	*p*-Value
Age, mean (SE)	39.8 (0.5)	38.2 (0.5)	37.9 (0.5)	0.05
Women, *n* (%)	1336 (51.6)	445 (55.1)	844 (52.8)	0.24
Race/ethnicity, *n* (%)				<0.0001
Non-Hispanic White	1101 (62.9)	235 (41.3)	531 (44.0)	
Non-Hispanic Black	511 (12.4)	189 (20.4)	357 (21.2)	
Hispanic	832 (16.9)	365 (31.9)	625 (29.3)	
Other race/ethnicity	155 (7.8)	30 (6.4)	74 (5.5)	
Educational attainment, *n* (%)				<0.0001
<12 years	793 (23.1)	313 (32.8)	728 (40.6)	
High school diploma or equivalent	667 (26.4)	220 (30.3)	402 (27.0)	
Any college	756 (31.6)	202 (26.5)	390 (27.6)	
College graduate or higher	383 (19.0)	84 (10.4)	67 (4.8)	
Household income (as ratio to federal poverty level), mean (SE)	1.68 (0.02)	1.38 (0.03)	1.15 (0.03)	<0.0001
Marital status, *n* (%)				0.0009
Married or living with partner	1475 (57.4)	457 (53.2)	819 (51.5)	
Never married	637 (26.8)	192 (25.2)	388 (26.4)	
Separated, divorced, or widowed	487 (15.8)	170 (21.7)	80 (22.0)	

**Table 2:** Household food security and biomarkers of cumulative biological risk: National Health and Nutrition Examination Surveys 2007–2010.

	Food-Secure	Marginal Food Secure	Food-Insecure	*p*
Systolic blood pressure				
Mean (SE)	118.7 (0.4)	118.9 (0.6)	118.1 (0.6)	0.52
Low-risk (<120 mmHg), *n* (%)	1212 (52.2)	352 (51.0)	753 (56.6)	0.04
Moderate-risk (120–<150 mmHg), *n* (%)	692 (28.6)	238 (31.7)	383 (24.3)	
High-risk (≥120 mmHg), *n* (%)	535 (19.2)	160 (17.3)	317 (19.1)	
Diastolic blood pressure				
Mean (SE)	70.8 (0.6)	70.7 (0.5)	70.7 (0.6)	0.99
Low-risk (<80 mmHg), *n* (%)	1597 (67.4)	502 (69.6)	954 (68.9)	0.69
Moderate-risk (80–<90 mmHg), *n* (%)	288 (12.0)	87 (12.3)	178 (11.6)	
High-risk (≥90 mmHg), *n* (%)	554 (20.6)	161 (18.0)	321 (19.5)	
Body mass index (kg/m^2^)				
Mean (SE)	28.8 (0.2)	29.6 (0.3)	29.4 (0.3)	0.06
Low-risk (<25 kg/m^2^), *n* (%)	770 (32.8)	216 (29.0)	420 (29.5)	0.19
Moderate-risk (25–<30 kg/m^2^), *n* (%)	829 (31.4)	267 (30.7)	505 (31.7)	
High-risk (≥30 kg/m^2^), *n* (%)	972 (35.7)	328 (40.3)	648 (38.8)	
Glycohemoglobin				
Mean (SE)	5.53% (0.03%)	5.57% (0.04%)	5.66% (0.03%)	0.01
Low-risk (<5.7%), *n* (%)	1664 (72.6)	497 (67.7)	975 (68.8)	0.18
Moderate-risk (5.7–<6.5%), *n* (%)	522 (19.2)	198 (23.0)	360 (21.8)	
High-risk (≥6.5%), *n* (%)	278 (8.2)	90 (9.3)	181 (9.4)	
Total cholesterol (mg/dL)				
Mean (SE)	196.3 (1.0)	193.1 (1.6)	196.5 (1.8)	0.23
Low-risk (<200 mg/dL), *n* (%)	1277 (53.4)	418 (56.9)	792 (54.2)	0.38
Moderate-risk (200–<240 mg/dL), *n* (%)	680 (26.9)	222 (27.7)	424 (27.3)	
High-risk (≥240 mg/dL), *n* (%)	495 (19.7)	139 (15.4)	293 (18.5)	
HDL cholesterol (mg/dL)				
Mean (SE)	51.0 (0.5)	50.0 (0.8)	49.8 (0.6)	0.07
Low-risk (≥60 mg/dL), *n* (%)	628 (26.2)	177 (23.0)	334 (21.6)	0.05
Moderate-risk (40–<60 mg/dL), *n* (%)	1207 (48.8)	377 (48.0)	775 (52.1)	
High-risk (<40 mg/dL), *n* (%)	608 (25.0)	221 (29.0)	398 (26.3)	
Total/HDL cholesterol ratio				
Mean (SE)	4.21 (0.04)	4.26 (0.08)	4.34 (0.06)	0.1
Low-risk (<5), *n* (%)	1838 (76.0)	567 (73.0)	1103 (73.1)	0.24
Moderate-risk (5–<6), *n* (%)	313 (11.9)	98 (12.7)	187 (11.9)	
High-risk (≥6), *n* (%)	292 (12.1)	110 (14.3)	217 (15.0)	
C-reactive protein				
Mean (SE)	0.37 (0.01)	0.46 (0.03)	0.46 (0.03)	0.005
Low-risk (<1 mg/L), *n* (%)	2197 (90.5)	691 (89.5)	1319 (87.8)	0.12
Moderate-risk (1–<3 mg/L), *n* (%)	232 (8.5)	81 (9.7)	164 (10.5)	
High-risk (≥3 mg/L), *n* (%)	25 (1.0)	8 (0.8)	29 (1.6)	
Albumin				
Mean (SE)	4.29 (0.01)	4.28 (0.02)	4.22 (0.02)	0.0002
Low-risk (≥3.8 mg/mL), *n* (%)	2302 (95.8)	731 (94.1)	1380 (92.3)	*n*/A
Moderate-risk (3.0–<3.8 ug/mL), *n* (%)	129 (4.2)	41 (5.9)	115 (7.0)	
High-risk <3.0 mg/mL), *n* (%)	2 (0.1)	0	8 (0.7)	
Estimated Glomerular Filtration Rate (mL/min)				
Mean (SE)	101.1 (0.6)	102.6 (0.9)	102.2 (0.8)	0.3
Low-risk (≥60 mL/min/1.73 m^2^), *n* (%)	2378 (98.5)	751 (97.5)	1455 (97.4)	0.08
Moderate-risk (30–<60 mL/min/1.73 m^2^), *n* (%)	45 (1.3)	16 (2.0)	39 (2.3)	
High-risk (<30 mL/min/1.73 m^2^), *n* (%)	10 (0.2)	5 (0.5)	8 (0.4)	

**Table 3:** Associations between household food security and cumulative biological risk: National Health and Nutrition Examination Surveys 2007–2010.

	Cumulative Biological Risk Score				Elevated Biological Risk (Score ≥ 3)			
	Age- and Gender-Adjusted		Multivariable-Adjusted		Age- and Gender-Adjusted		Multivariable-Adjusted	
	β	95% CI	β	95% CI	OR	95% CI	OR	95% CI
All adults								
Food secure	Ref.	-	Ref.	-	Ref.	-	Ref.	-
Marginal food secure	0.18	−0.02, 0.38	0.13	−0.05, 0.30	1.26	0.97, 1.64	1.22	0.97, 1.55
Food insecure	0.22	0.11, 0.32	0.14	0.05, 0.22	1.27	1.10, 1.47	1.20	1.05, 1.37
*p*-trend	0.0002		0.003		0.0003		0.003	
Men								
Food secure	Ref.	-	Ref.	-	Ref.	-	Ref.	-
Marginal food secure	0.13	−0.21, 0.47	0.07	−0.25, 0.39	1.25	0.77, 2.02	1.16	0.72, 1.88
Food insecure	0.02	−0.17, 0.22	−0.02	−0.20, 0.15	0.98	0.75, 1.28	0.93	0.72, 1.20
*p*-trend	0.72		0.86		0.95		0.70	
Women								
Food secure	Ref.	-	Ref.	-	Ref.	-	Ref.	-
Marginal food secure	0.23	0.04, 0.42	0.18	0.01, 0.35	1.30	0.94, 1.80	1.31	0.95, 1.79
Food insecure	0.41	0.24, 0.58	0.30	0.14, 0.45	1.71	1.39, 2.10	1.61	1.29, 2.00
*p*-trend	<0.0001		0.0004		<0.0001		<0.0001	

Multivariable model further adjusted for race/ethnicity, educational attainment, household income, and marital status. *p*-interaction for multivariable-adjusted model of cumulative biological risk by gender was 0.09, and for multivariable-adjusted model of elevated biological risk by gender was 0.03.

## Supplementary Materials

The following are available online at https://www.mdpi.com/2072-6643/12/5/1517/s1, Table S1: Associations between household food security (using the four-category variable) and cumulative biological risk: National Health and Nutrition Examination Surveys 2007–2010.
