# Supplementary material for: Household Food Insecurity and the Association with Cumulative Biological Risk among Lower-Income Adults: Results from the National Health and Nutrition Examination Surveys 2007–2010
Source: Nutrients. 2020 May 23;12(5):1517. doi: 10.3390/nu12051517 (PMC7285083; doi:10.3390/nu12051517)
Supplement: Supplementary file 1 [file nutrients-12-01517-s001.pdf]

Supplemental Table S1: Associations between household food security (using the four-category variable) and cumulative biological risk: National Health and Nutrition Examination Surveys 2007-2010

	Cumulative biological dysregulation score				Elevated biological dysregulation (score ≥ 3)			
	Age- and gender-adjusted		Multivariable-adjusted		Age- and gender-adjusted		Multivariable-adjusted	
		95% CI		95% CI	OR	95% CI	OR	95% CI
All adults								
Food secure	Ref.	-	Ref.	-	Ref.	-	Ref.	-
Marginal food secure	0.18	-0.02, 0.38	0.13	-0.05, 0.30	1.24	0.96, 1.59	1.22	0.97, 1.55
Low food security	0.25	0.11, 0.39	0.16	0.04, 0.28	1.27	1.04, 1.55	1.20	0.98, 1.46
Very low food security	0.16	0.01, 0.31	0.09	-0.06, 0.23	1.24	1.01, 1.53	1.20	0.98, 1.49
P-trend	*0.0005*		*0.008*		*0.0006*		*0.004*	
Men								
Food secure	Ref.	-	Ref.	-	Ref.	-	Ref.	-
Marginal food secure	0.13	-0.21, 0.47	0.08	-0.25, 0.39	1.25	0.77, 2.02	1.16	0.72, 1.88
Low food security	0.07	-0.16, 0.29	0.01	-0.20, 0.23	1.03	0.73, 1.46	0.98	0.69, 1.38
Very low food security	-0.06	-0.29, 0.16	-0.09	-0.30, 0.11	0.89	0.66, 1.20	0.86	0.66, 1.13
P-trend	*0.9*		*0.67*		*0.85*		*0.5*	
Women								
Food secure	Ref.	-	Ref.	-	Ref.	-	Ref.	-
Marginal food secure	0.23	0.04, 0.42	0.18	0.01, 0.35	1.30	0.94, 1.80	1.31	0.95, 1.79
Low food security	0.43	0.23, 0.63	0.31	0.11, 0.51	1.64	1.26, 2.15	1.53	1.14, 2.05
Very low food security	0.37	0.11, 0.63	0.28	0.03, 0.53	1.82	1.28, 2.59	1.74	1.21, 2.49
P-trend	*<0.0001*		*0.001*		*<0.0001*		*<0.0001*	

Multivariable model further adjusted for race/ethnicity, educational attainment, household income, and marital status. P-interaction for multivariable-adjusted model of cumulative allostatic load by gender was 0.08, and for multivariable-adjusted model of elevated allostatic load by gender was 0.02.
